# A paradigm shift and practical exploration in veterinary pharmacology and toxicology teaching reform in China’s frontier regions under AI empowerment

**DOI:** 10.3389/fvets.2026.1736521

**Published:** 2026-02-18

**Authors:** Meiquan Li, Yuru Ma, Yanli Du, Xiao Wang, Hongyan Zhang

**Affiliations:** 1 College of Agriculture and Life Sciences, Kunming University, Kunming, China; 2 Reproductive Genetics Department, The First Affiliated Hospital of Kunming Medical University, Kunming, China

**Keywords:** artificial intelligence, high-altitude animal diseases, southwest border region, teaching reform, veterinary pharmacology and toxicology

## Abstract

Veterinary pharmacology and toxicology education in Southwest China’s border regions faces unique challenges, including plateau animal diseases and transnational epidemic control, and the need for strict veterinary drug residue monitoring and toxic risk prevention, which traditional teaching methods cannot adequately address. This study explores an AI-driven educational reform centered on data-driven instruction and virtual reality integration. By constructing dynamic regional knowledge graphs, personalized learning pathways, and high-fidelity simulation platforms for plateau pharmacology experiments, clinical decision-making, and toxicological risk assessment, we establish an intelligent teaching system tailored to frontier characteristics. The system deeply integrates pharmacological efficacy research with toxicological safety evaluation, an integration emphasized as the cornerstone of veterinary practice in ensuring drug safety, controlling residue risks, and safeguarding the “One World, One Health” paradigm. The reform also addresses digital divide risks while developing a multidimensional evaluation framework covering both therapeutic effectiveness and toxicological safety competencies. This paradigm shift aims to enhance the quality of veterinary pharmacology and toxicology education in border regions by providing systematic solutions for cultivating professionals capable of balancing drug efficacy and safety, addressing regional toxicological challenges, and serving regional development needs.

## Introduction

The primary objective of veterinary pharmacology and toxicology education is to equip students with a comprehensive, translational understanding of drug action and safety in animals, enabling them to make safe, effective, economical, and ethically sound decisions regarding drug use in complex clinical and environmental settings (1). This integrated perspective is fundamental to the “One World, One Health” paradigm, as it directly addresses risk mitigation at the animal–human–ecosystem interface (2). While this objective is consistently upheld across the nation, unique challenges emerge when focusing on China’s southwestern frontier regions. These areas exhibit distinct socioeconomic, geographic, and epidemiological characteristics that impose specific demands on veterinary pharmacology and toxicology instruction—demands that differ significantly from those in inland and more developed regions (3).

### The unique pharmacological and toxicological considerations associated with treating diseases in plateau-dwelling animals

The high-altitude environment, characterized by hypoxia, intense ultraviolet radiation, and distinct vegetation, significantly influences the physiological status and pharmacokinetic profiles of animals. For example, drug absorption, distribution, metabolism, and excretion in these animals may be markedly altered, potentially increasing the risk of adverse effects or therapeutic failure (4). Conventional dosing regimens derived from lowland studies may therefore result in suboptimal outcomes or unexpected toxicities in high-altitude settings (5). This underscores the need for an integrated pharmacology-toxicology perspective in the curriculum, in which understanding drug efficacy (pharmacology) is inseparable from predicting and managing its potential toxicity (toxicology), particularly under unique physiological stressors (2).

### The critical need for cross-border disease prevention and control

Border regions are particularly vulnerable to the introduction and spread of major transboundary animal diseases, such as foot-and-mouth disease and African swine fever (6). Veterinary professionals must be equipped with the capability to rapidly identify, diagnose, and implement appropriate pharmacological interventions for emergency response and disease containment. This necessitates that educational content be highly timely, practical, and aligned with real-world field demands, including the assessment of drug residues and environmental impacts (7).

### The practical requirements of regional animal husbandry development

**Figure 1:**
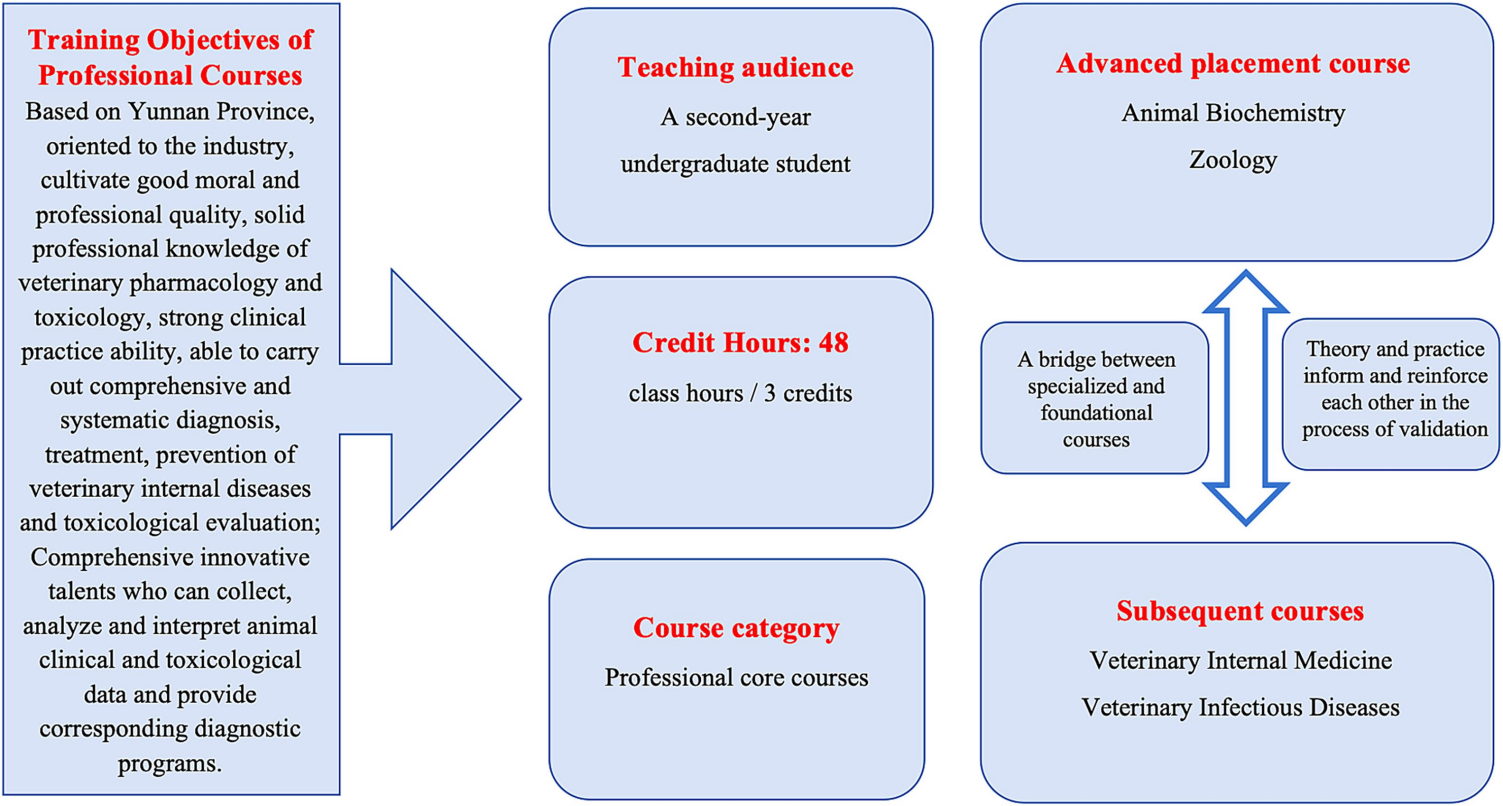
Fundamental information for veterinary pharmacology and toxicology courses.

Veterinary pharmacology and toxicology instruction must align with and support local dominant industries. A key challenge lies in training students to design cost-effective, efficient, and food safety-compliant drug use strategies tailored to the predominant livestock species and integrated breeding systems—both traditional and modern—prevalent in the region (8, 9). This training is essential for cultivating veterinary professionals who are capable of working effectively at the grassroots level, contributing meaningfully, and remaining committed to rural service. The structural constraint is posed by uneven educational resource distribution. Compared to central and eastern regions of China, southwestern border areas face significant shortages in veterinary education resources and high-quality clinical training facilities. Limited access to advanced knowledge and exposure to complex clinical cases hinders both faculty and students, thereby widening the gap between theoretical instruction and practical application (10). Based on the above analysis, the unique demands of veterinary education in the southwestern border regions fundamentally necessitate a teaching paradigm that deeply integrates pharmacology and toxicology. This integration is not only essential for enhancing therapeutic efficacy but also serves as the cornerstone for ensuring the safety of animal-derived foods, preventing and controlling cross-border public health risks, and safeguarding frontier ecological stability. Consequently, the core of this teaching reform is to construct a new educational system aimed at fostering “efficacy-safety synergistic competency,” supported by intelligent technologies (Figure 1).

### The development of AI technology and national strategic deployment

As the aforementioned challenges become increasingly pronounced, national-level strategic initiatives to advance the construction of new agricultural sciences and cultivate outstanding veterinary professionals have created a favorable policy environment for educational reform (11). Concurrently, the rapid advancement and growing maturity of technologies such as artificial intelligence, big data, cloud computing, and virtual reality have provided unprecedented technological means to address long-standing issues in border regions, including disparities in educational resources and insufficient access to practical training scenarios (12). Consequently, this phase of reform represents not merely an incremental integration of technological tools but a fundamental transformation in the educational paradigm. The core of this shift lies in transitioning from traditional knowledge transmission to future-oriented capability empowerment (12); from teacher-centered instruction to personalized, student-driven exploration facilitated by human–machine collaboration; and from standardized talent development to regionally tailored, precision-based education. This study aims to systematically elucidate the underlying logic, practical pathways, and prospects of this paradigmatic transformation (3).

### In-depth analysis of the core challenges in traditional veterinary pharmacology and toxicology education in border regions

Through an in-depth analysis of teaching feedback and learning data collected from multiple agricultural colleges and universities in the southwestern border region, the primary challenges of the traditional teaching model in this area can be summarized into the following three aspects:

### Dilemma one: the mismatch between universal textbooks and regional knowledge requirements

The incompatibility of the knowledge system. Although nationally standardized textbooks feature a comprehensive structure, their content predominantly focuses on lowland regions and common livestock species, lacking systematic coverage of plateau animal physiology and pharmacology, the epidemiological profiles of region-specific diseases, and drug applications for transboundary animal diseases (13). This results in a significant gap between academic training and post-graduation professional demands, leading to the prevalent issue of “what is learned cannot be applied, and what is needed is not taught.” Consequently, students are unable to develop the cognitive frameworks necessary for addressing local challenges during their on-campus education.

### Dilemma two: the disconnect between static theoretical instruction and dynamic clinical decision-making

Deficiencies in medication-thinking training in high-altitude contexts. When presented with a case of respiratory disease in calves on a high-altitude ranch, students tend to apply textbook-recommended antibacterial agents without adequately considering how high-altitude conditions may influence drug metabolism rates, tissue distribution, and the consequent necessity for dosage adjustments (14). Their understanding of species-specific factors, such as contraindications related to rumen function in ruminants, remains superficial (15).

Gaps in dynamic decision-making training for transboundary disease control. In simulated scenarios involving the introduction of cross-border epidemics, students struggle to implement a sequence of dynamic responses, including risk assessment, quarantine measures, therapeutic interventions, and surveillance. Conventional teaching methods fail to replicate rapidly evolving, multifactorial emergencies, thereby limiting the development of students’ practical competencies (16, 17).

### Dilemma three: the misalignment between a single-dimensional evaluation system and comprehensive professional competence

Inability to assess regional problem-solving capabilities. Standardized written examinations are inherently limited in evaluating students’ holistic skills, such as designing effective deworming protocols for alpine pastures, assessing the economic viability and cultural acceptability of specific pharmaceuticals in ethnically distinct communities, or formulating community-level medication strategies for disease prevention in border villages (18, 19).

Insufficient cultivation of commitment to serving border regions. The curriculum exhibits weak integration with national strategic priorities such as border-area animal husbandry development, rural revitalization, and national biosecurity. As a result, students demonstrate diminished recognition of professional value and a weakened sense of mission toward veterinary practice in remote border areas, which undermines both their intrinsic motivation to learn and their long-term willingness to serve these underserved regions.

### The core path of teaching reform in China’s border regions driven by artificial intelligence and digital technologies

In response to the aforementioned challenges, the reform driven by AI technology has systematically conducted practice-oriented explorations with distinctive boundary-crossing features across three dimensions: reconstruction of teaching content, reengineering of the teaching process, and innovation in teaching evaluation (20).

### Reconstruction of teaching content: from universal textbooks to dynamic and regionalized knowledge networks

Construction of a dynamic knowledge graph with border characteristics: By utilizing natural language processing and knowledge graph technologies, this approach integrates key resources, including the physiological databases of plateau animals, disease profiles, and medication guidelines for animals in border regions, cross-border disease surveillance and control strategies, regional standards for veterinary drug residue limits, and cutting-edge domestic and international research literature (21). This integration enables the development of a specialized, dynamic knowledge base in veterinary pharmacology and toxicology tailored to the southwest border regions. The resulting knowledge graph visually represents the complex interrelationships among “drugs–targets–plateau physiology–endemic diseases–cross-border risks–residue standards” through a networked structure. For example, a query on ivermectin not only displays its general pharmacological properties but also highlights its metabolic behavior in high-altitude yaks, its application protocols for prevalent local parasitic diseases, and potential interactions with traditional herbal remedies commonly used in specific ethnic communities (22, 23).

Modular and Granular Regional Curriculum Design. The curriculum content is restructured into three core modules: Core Pharmacology Module (Fundamental and General): This module covers the foundational principles of pharmacodynamics and pharmacokinetics. Frontier Characteristic Pharmacology Module (Specialized and Context-Specific): This module provides an in-depth exploration of pharmacological aspects related to plateau-adapted animals, treatment protocols for cross-border transmissible diseases, livestock and poultry pharmacology in ethnic minority regions, and the management of veterinary drug residues and food safety in frontier zones (24). Crucially, it integrates toxicological perspectives, including mechanisms of drug-induced toxicity in high-altitude environments, residue-related risk assessment, and the management of adverse drug reactions in unique regional contexts, ensuring a holistic understanding of drug efficacy and safety. Specialized Application Modules (Skill Enhancement): Examples include “Strategies for Antibiotic Resistance Management in High-Altitude Pastoral Areas” (24), “Emergency Medication Drills for Borderline Animal Epidemics” (25), and “Safe Medication Use During the Peripartum Period for Specialty Economic Animals Such as Tibetan Pigs and Forest-Grown Free-Range Chickens” (26).

### Reengineering the teaching process: from one-way lecture to human–machine collaborative, personalized, and immersive learning

Adaptive learning systems and intelligent navigation. The AI platform generates a “competency profile” for each student through preliminary assessments and continuous learning behavior analysis (27). For students aspiring to work at grassroots veterinary stations, the system intelligently recommends more content on common disease prevention and group medication. For those aiming to engage in border quarantine, it enhances training on cross-border epidemic medication decision-making and associated toxicological vigilance, achieving true “region-specific teaching.”

Virtual pharmacology experiments and an integrated pharmacology–toxicology simulator for plateau animals: We developed a virtual experimental platform capable of simulating environments at various altitudes (e.g., from 500 m to 4,500 m). On this platform, students can observe differences in drug–time curves for a specific antibiotic in simulated “highland Tibetan sheep” vs. “plains sheep,” explore correlation with pharmacodynamic effects, and gain an intuitive understanding of the scientific rationale for adjusting dosing regimens in plateau environments (28–30). Reliable *in vitro* toxicity assays that mimic *in vivo* physiological phenotypes are indispensable for understanding toxic mechanisms and ensuring pharmaceutical safety, complementing and refining animal testing where practical (31). This alignment with rigorous toxicological testing principles enables the virtual platform to replicate key processes, such as drug metabolic toxicity and residue accumulation, in plateau-adapted animals, equipping students with the critical ability to associate pharmacological efficacy with toxicological safety in region-specific contexts.

Intelligent virtual case database (Frontier Edition). AI generates massive dynamic cases based on real-world data from the southwestern frontier, such as mixed high-altitude sickness of *Escherichia coli* in calves in high-altitude areas, suspected outbreaks of foreign diseases in pig herds in border villages, and prevention and control of coccidiosis in chickens raised in forests (32–34). Students managing virtual patients are required to complete the full workflow—from assessment of environmental factors to diagnosis and prescription—incorporating mandatory considerations of drug toxicity profiles, prediction of potential adverse effects, and design of residue monitoring plans. The AI system functions as an intelligent mentor, providing real-time feedback on both therapeutic efficacy and toxicological appropriateness (35, 36).

AR/VR-enabled immersive border scenes. Through VR technology, students can “immerse themselves” in high-altitude pastures, border quarantine stations, or breeding farms in ethnic minority villages for virtual consultations and medication guidance, greatly enhancing the sense of presence and immersion in the learning process (37–39).

Digital twin border farm and one health practice. Building a Virtual Farm Linked to Real Border Farm Data. Students serve as virtual on-site veterinarians to develop year-round plans for deworming, immunization, and antibiotic use, and observe the long-term impact of these plans on the production efficiency of virtual farms, drug residues in the surrounding environment, and changes in bacterial resistance. They deeply understand the enormous responsibility of veterinarians in maintaining animals’ health in border areas where pharmacological efficacy must be continuously balanced with toxicological safety across the animal–human–environment interface (2, 40).

### Innovation in teaching evaluation: from result scoring to process- and ability-oriented intelligent evaluation

Digital portrait evaluation through multi-source data fusion. The system comprehensively records students’ performance across multiple scenarios, such as regional case studies, virtual plateau experiments, and cross-border epidemic decision simulations, and generates a comprehensive and process-based evaluation of their knowledge, skills, attitudes, and regional thinking process (41, 42).

Analysis of an AI-driven regional clinical thinking process. In the virtual case assessment, the AI evaluates students’ decision-making logic when dealing with frontier-specific characteristics: Are environmental factors considered? Has cross-border risk been assessed? Does the medication plan conform to the local economic conditions and breeding practices? The system generates a detailed “Regional Clinical Thinking Assessment Report” and accurately identifies areas where students’ reasoning thinking blind may be incomplete or flawed (43).

The frontier microcertificate system is enabled by blockchain technology. Students receive a blockchain-based digital microcertificate upon completing ability modules with frontier-specific characteristics, such as “expert on safe drug use for plateau animals”, or “cross-border epidemic drug emergency responder”, and successfully passing the associated challenge. These certificates have become authoritative proof of their capability to serve frontier settings, enhancing both their employment competitiveness and social recognition, and signaling their dual competence in ensuring therapeutic efficacy and comprehensive drug safety (44, 45).

### Special challenges and ethical considerations faced by reform in the border area

#### Realistic challenges

Digital divide and education equity: Due to the uneven coverage of network infrastructure and limited bandwidth in border areas, some grassroots colleges and universities find it difficult to smoothly operate high-standard AI teaching platforms (46, 47). The high cost of investment and maintenance of hardware equipment may exacerbate the inequality of educational resources between schools and regions (48).

Multilingualism and cultural adaptation are needed. The southwestern frontier is a multi-ethnic area, and there is a need to develop auxiliary teaching resources for minority languages such as Yi and Tibetan. At the same time, the AI model and case design should fully consider the local cultural practices, traditional breeding knowledge, and medication habits to prevent cultural conflicts (49).

Regional data barriers and standardization dilemma. Constructing a high-quality regional knowledge map depends on a large amount of standardized, localized data. However, the data on animal disease monitoring, drug use records, residue detection, and other related datasets in border areas are often scattered in different departments, with different standards and an imperfect sharing mechanism (50, 51).

Teachers’ role transformation and digital literacy improvement. Teachers in border areas may be more familiar with the traditional teaching mode and face challenges in their role transformation to learning designers and human–computer collaborative guides, requiring systematic digital skills training and continuous teaching support (52).

#### Ethical considerations

Data privacy and security. To collect students’ learning process data and regional practice data, we must strictly abide by privacy protection laws and regulations to ensure data security and prevent information leakage (53, 54).

Algorithm bias and fairness. The training data of the AI model must fully represent the diversity of border areas, prevent algorithm discrimination against specific breeding modes, species, or ethnic groups, and ensure the fairness of evaluation and recommendation (55).

Balance between technology dependence and humanistic spirit. It is necessary to remain vigilant that overreliance on virtual simulation may weaken students’ basic practical ability, such as the lack of experience in animal restraint, the lack of injection-handling techniques, and the weakening of empathy and humanistic care when communicating with border herdsmen and farmers. Technology is an enabling tool, which can never replace the reverence for life, the love for the countryside, and the deep feelings with the people.

### Future prospects: building a new ecology of frontier-intelligent veterinary pharmacology and toxicology education

In-depth application of generative AI. In the future, generative AI can be used to generate highly customized teaching cases and assessment questions in real-time according to the specific animal husbandry structure, disease prevalence, and resource conditions of any county or city on the southwest frontier of China. This includes the simulation of complex toxicological scenarios, such as managing mass drug poisoning incidents or predicting residue carryover in diverse farming systems, thereby providing an infinite approximation to real speculative training (56, 57).

Constructing a cross-regional and inter-university “virtual frontier teaching and Research Office”. This initiative promotes the establishment of a virtual teaching and research organization led by colleges and universities in the frontier region, with participation from high-level inland universities and research institutes. Through the joint development and sharing of the frontier characteristic teaching resource library, case library, and AI algorithm model, a collaborative development community can be formed (58).

Developing a lightweight, mobile-first intelligent teaching platform: According to the characteristics of the border network, a lightweight app should be developed that can be used smoothly in the mobile terminal offline or in a low-bandwidth environment, extending intelligent teaching to pasture, field, and grass-roots veterinary stations and enabling access to ubiquitous learning “anytime, anywhere” (59).

**Figure 2:**
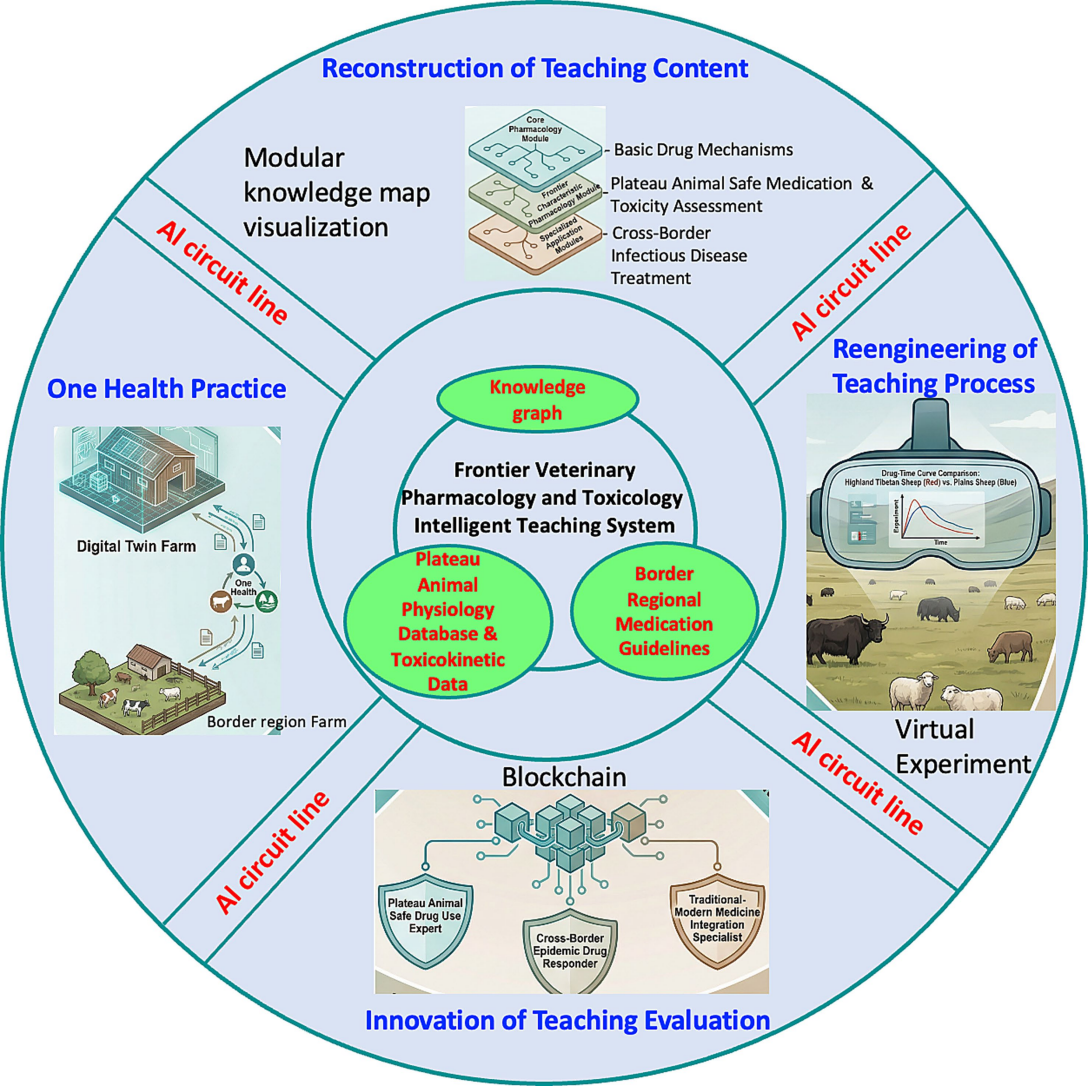
Reconstruction of teaching content in the Frontier Veterinary Pharmacology and Toxicology Intelligent Teaching System. This infographic illustrates the four-pillar framework for reconstructing teaching content: (1) Core Knowledge Modules (top circle), integrating basic pharmacology and toxicology with frontier-specific content; (2) Knowledge Graph & Data Foundation (center), serving as the system’s “brain” with data from plateau physiology, toxicokinetic data, regional guidelines, and traditional-modern medicine integration; (3) Virtual Experiments and Comparative Analysis (right), featuring a drug-time curve comparison between highland Tibetan sheep and plains sheep; and (4) Digital Twin Farm and One Health Integration (left), connecting virtual simulations with real-world frontier farm scenarios. The bottom banner highlights that this content reconstruction ultimately supports innovation in teaching evaluation. The design emphasizes the interconnectedness of modular knowledge, data-driven intelligence, virtual-real integration, and frontier-specific applications.

AI-assisted frontier drug R&D enlightenment. At the advanced stage, an AI drug design simulation platform can be introduced to enable excellent students to experience the initial process, from target discovery to lead compound optimization for unique pathogens or drug-resistant bacteria in frontier areas, thereby sowing seeds for the cultivation of veterinary scientists rooted in the frontier in the future (Figure 2).

## Conclusion

The teaching reform of veterinary pharmacology and toxicology, marked by AI and digital technology, is triggering a profound change in the education concept, teaching mode, and evaluation system in the southwest frontier region of China. The essence of this change is to build a new education ecology centered on the comprehensive development of learners, driven by data intelligence, supported by the integration of virtual and real, and deeply rooted in the frontier land. It aims to transform students from passive recipients of universal knowledge into future veterinary professionals who, with the support of intelligent systems, can actively integrate pharmacological efficacy with toxicological safety, explore frontier-specific problems, innovate regional solutions, and take responsibility for the sustainable development of frontier animal husbandry, food safety, and national biosecurity. Ultimately, this reform cultivates a new generation of practitioners who are not only therapeutic experts but also guardians of drug safety, equipped to navigate the complex interplay between cure and care, efficacy and risk, in some of the most challenging and vital environments for veterinary medicine.
